# Recent Advances in the Transcriptional Regulation of Secondary Cell Wall Biosynthesis in the Woody Plants

**DOI:** 10.3389/fpls.2018.01535

**Published:** 2018-10-23

**Authors:** Jin Zhang, Meng Xie, Gerald A. Tuskan, Wellington Muchero, Jin-Gui Chen

**Affiliations:** ^1^Biosciences Division, Oak Ridge National Laboratory, Oak Ridge, TN, United States; ^2^Center for Bioenergy Innovation, Oak Ridge National Laboratory, Oak Ridge, TN, United States; ^3^Department of Plant Sciences, University of Tennessee, Knoxville, TN, United States

**Keywords:** woody plants, *Populus*, secondary cell wall, transcription factor, transcriptional regulation

## Abstract

Plant cell walls provide structural support for growth and serve as a barrier for pathogen attack. Plant cell walls are also a source of renewable biomass for conversion to biofuels and bioproducts. Understanding plant cell wall biosynthesis and its regulation is of critical importance for the genetic modification of plant feedstocks for cost-effective biofuels and bioproducts conversion and production. Great progress has been made in identifying enzymes involved in plant cell wall biosynthesis, and in *Arabidopsis* it is generally recognized that the regulation of genes encoding these enzymes is under a transcriptional regulatory network with coherent feedforward and feedback loops. However, less is known about the transcriptional regulation of plant secondary cell wall (SCW) biosynthesis in woody species despite of its high relevance to biofuels and bioproducts conversion and production. In this article, we synthesize recent progress on the transcriptional regulation of SCW biosynthesis in *Arabidopsis* and contrast to what is known in woody species. Furthermore, we evaluate progress in related emerging regulatory machineries targeting transcription factors in this complex regulatory network of SCW biosynthesis.

## Introduction

Trees are important natural sources of sustainable energy and have important ecological and economical values (Tuskan, 1998; Richmond, 2000; Ragauskas et al., 2014). The majority of biomass of trees resides in the wood of stems, branches and roots. Wood is the major product of secondary growth derived from a lateral meristem, i.e., the vascular cambium, which forms xylem inwards and phloem outwards (Zhang et al., 2015). Prior to forming specialized cell types, cells in xylem and phloem undergo cell expansion and primary cell wall biosynthesis. However, wood is primarily composed of secondary cell walls (SCW) (Sundell et al., 2017). As the most abundant plant biomass worldwide, wood and fibers are widely used for various industrial applications, such as energy, pulping and textiles. In xylem, all the cell type firstly undergo SCW thickening and lignification, after which vessel elements and fibers undergo programmed cell death (PCD) (Courtois-Moreau et al., 2009).

Secondary cell walls, composed of lignin, cellulose and hemicelluloses, play an important role in plant development and stress responses (Houston et al., 2016). The maturation of SCWs reinforces specialized cells such as fibers and vessels, allowing them form mechanical tissues to provide structural support and protection while enabling negative pressure gradients generated during transpiration (Zhong et al., 2010a). The formation of SCW is a complex process requiring coordination of several metabolic pathways. Understanding the regulatory mechanism controlling SCW formation is critical for providing molecular and genetic basis for industrial applications (Zhong et al., 2013).

To date, a regulatory network consisting of several different types of transcription factors (TFs) and controlling SCW formation in the model plant *Arabidopsis* has been constructed (Zhong et al., 2010a; Taylor-Teeples et al., 2015). Recently, Rao and Dixon (2018) compared the transcriptional regulation models of SCW biosynthesis in grasses and *Arabidopsis*, and showed that the regulatory network of SCW development in grasses is relatively conserved with divergences. Compared to the annual herbaceous *Arabidopsis* and grasses, perennial woody species display extreme secondary growth that undergo seasonal changes that are impacted by various environmental stresses. Wood formation in perennial woody species is a dynamic and continuous process, which includes cambial cell proliferation, xylem cell differentiation, SCW thickening and PCD (Zhang et al., 2014). A comprehensive transcriptional regulatory network controlling secondary cell wall formation in woody species is still lacking. This review synthesizes the current advances of SCW regulatory network in plants in general and aims to highlight the recent progresses in this area in woody species. We also discuss the direction for future research in woody species.

## The First Layer of Transcription Factors in the Regulatory Network in SCW Formation

**FIGURE 1:**
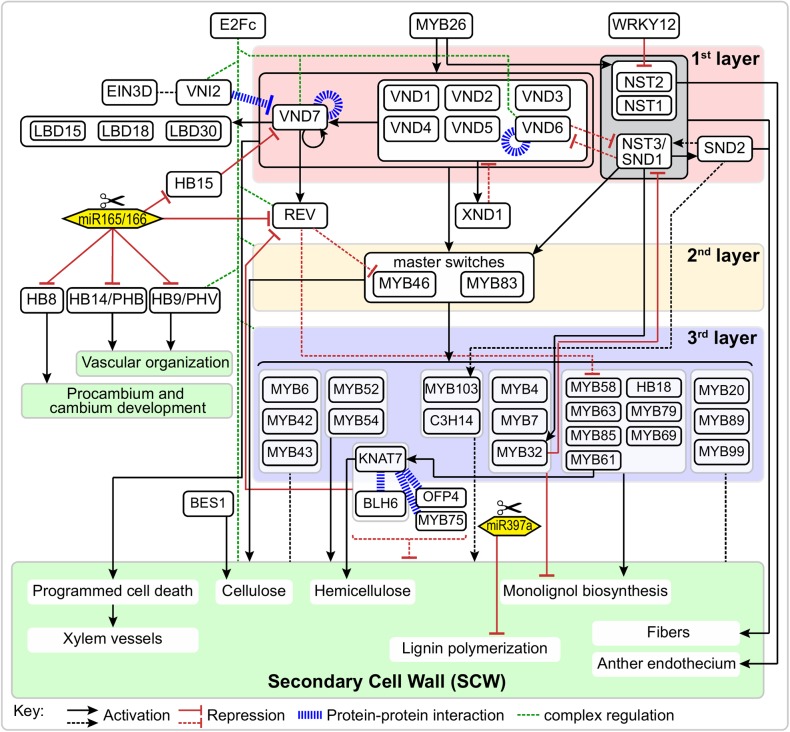
Schematic representation of the regulatory network of secondary cell wall formation. Arrows and “T” at the ends of lines represent activation and repression, respectively. Blue bold dash lines represent protein-protein interactions. Solid and dotted lines represent direct or indirect regulation, respectively.

NAC (NAM, ATAF, and CUC) TFs are plant-specific transcriptional regulators and are widely involved in various biological processes, including growth/development and stress responses (Olsen et al., 2005). During SCW formation, a group of closely-related NAC TFs function as master switches, which were named SECONDARY WALL NACs (SWNs). In the first layer of the SCW regulatory network, SWNs are comprised of two types of NACs: VASCULAR-RELATED NAC DOMAINS (VNDs; VND1-7) and NAC SECONDARY WALL THICKENING PROMOTING FACTOR (NST)/SECONDARY WALL-ASSOCIATED NAC DOMAIN PROTEIN (SND) (NST1-3) (Figure 1). SWNs can bind to a 19 bp secondary wall NAC binding element (SNBE) sequences, (T/A)NN(C/T)(T/C/G)TNNNNNNNA(A/C)GN(A/C/T)(A/T), and directly activate the expression of downstream TFs in the second layer, as well as structural genes involved in SCW biosynthesis, cell wall modification, and PCD (Zhong et al., 2010c). In addition, a 11 bp tracheary element-regulating *cis*-element (TERE) [CT(T/C)NAA(A/C)GCN(A/T)] was identified through an *in vitro* tracheary element (TE) transdifferentiation study and was shown to be essential for TE-specific expression mediated by VNDs (Pyo et al., 2007; Ohashi-Ito et al., 2010; Yamaguchi et al., 2011).

The function of NACs in SCW formation was first reported in *Zinnia elegans*, in which a NAC TF *Z567* was found to be up-regulated during the transdifferentiation from mesophyll cell into TEs in an *in vitro* culture system (Demura et al., 2002). Subsequently, in *Arabidopsis* suspension cells, seven homologs of *Z567* were shown to be up-regulated during xylem vessel cell differentiation, which were named *VND1* through *VND7* (Kubo et al., 2005). *VNDs* individually display specific expression patterns and functions. For example, *VND1-5* are expressed in the vessels of stem, but not expressed in interfascicular fibers. Moreover, *VND4* and *VND5* are expressed in vessels of the root hypocotyl. Overexpressing *VND1-5* can activate the expression of TFs and structural genes involved in SCW biosynthesis and PCD (Zhou et al., 2014). *VND6* and *VND7* are specifically expressed in vessels, directing metaxylem and protoxylem vessel differentiation, respectively (Kubo et al., 2005). Different with the function of VNDs in vessels, NST1 and NST3/SND1 are master regulators of SCW biosynthesis in fibers (Mitsuda et al., 2007). In *Arabidopsis nst1-1 nst3-1* double mutant, the SCW thickening was completely suppressed in interfascicular fibers and secondary xylem without affecting the cell formation (Mitsuda and Ohme-Takagi, 2008). Similar to the functional redundancy of NST1 and NST3 in stem, NST1 and NST2 function redundantly in SCW formation in anthers (Mitsuda et al., 2005).

These master regulators have relatively conserved functions across plant species, though copy numbers vary by species. In *Medicago truncatula*, only one member, *MtNST1*, was identified corresponding to the three sequence homologs *NST1-3* in *Arabidopsis*. A loss-of-function mutant, *mtnst1*, results in reduced lignin and cell wall polysaccharide contents through regulating the expression of most lignin biosynthetic and cellulose and hemicellulose biosynthetic genes (Zhao et al., 2010). *Oryza sativa* secondary wall NAC domain protein 1 (OsSWN1), an ortholog of *Arabidopsis* NST3/SND1, also regulates SCW formation in rice (Zhong et al., 2011; Chai et al., 2015). In *Arabidopsis*, ectopic expression of *OsSWN1* induced massive ectopic deposition of lignified SCW in leaf mesophyll cells and in the epidermis and cortical cells of the inflorescence stems (Zhong et al., 2011). When *OsSWN1* was heterologously expressed driven by the *Arabidopsis NST3* promoter in the *nst1 nst3* double mutant, the pendent stem phenotype and the SCW lignification of inflorescent fibers were effectively rescued (Zhong et al., 2011), suggesting that OsSWN1 is functionally equivalent to *Arabidopsis* NST3/SND1. Subsequently, Sakamoto et al. (2016) overexpressed *OsSWN1* in poplar using *Arabidopsis NST3* promoter. The transgenic poplars displayed thickened SCW in xylem cells and phloem fiber cells but not in xylem vessels. A follow-up study indicated that overexpression of *OsSWN1* in *Populus* altered lignin structure, but not lignin content, due to an unbalance induction of lignin biosynthetic genes (Nuoendagula et al., 2017). This further confirms that the function of the master switches is conserved across different species, whether they are annual or perennial, herbaceous or woody. Consistent with this notion, the similar regulatory pathway is also observed in *Zea mays*, where *ZmNST3* and *ZmNST4* were specifically expressed in SCW-forming cells and functioned as master switches for SCW deposition through regulating the expression of *ZmMYB109/128/149* (Xiao et al., 2018).

**FIGURE 2:**
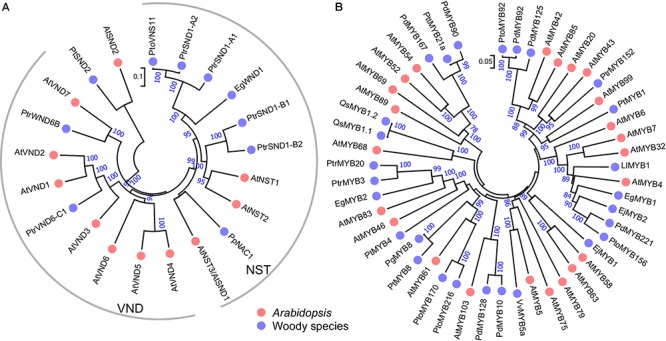
Phylogenetic trees of SWNs **(A)** and MYBs **(B)** in woody species and *Arabidopsis*. The unrooted phylogenetic trees were constructed with amino acid sequences of the known SWNs or MYBs in woody species from Table 1 and their homologous in *Arabidopsis* by the neighbor-joining method with 1000 bootstraps.

In woody species, similar master switches have also been identified in *Populus* and *Eucalyptus*. In *Populus*, a group of wood-associated NAC domain TFs, *PtrWNDs*, were identified as master transcriptional switches in SCW biosynthesis. Ohtani et al. (2011) isolated 16 *Populus* NAC TFs and designated them as *PtVNS* (VND-, NST/SND- and SMB-related)/*PtrWND*. Among them, 12 members in NST and VND groups are expressed in developing xylem and phloem fibers, whereas only the VND group members are expressed in primary xylem vessels. A homolog of *SND2* from *P. trichocarpa*, *PtSND2*, plays a similar role in the SCW biosynthesis. Chimeric repressor of *PtSND2* reduced the SCW thickness of xylem fibers and decreased lignin and cellulose contents in *Populus* (Wang et al., 2013). *PtrSND1-A2*/*PtrWND1B* (Potri.001G448400) was shown to be specifically expressed in secondary xylem fiber cells and suppression of *PtrWND1B* significantly inhibited fiber SCW thickening (Li Q. Z. et al., 2012; Zhao et al., 2014). Moreover, these master regulators function in gymnosperm trees. *Pinus pinaster PpNAC1* is a NST group TF, and it is a key regulator of phenylalanine biosynthesis through activating the expression of itself and *PpMYB4* (Table 1 and Figure 2) (Pascual et al., 2018). These results suggest that SWNs are ancestral master switches for the SCW formation, and that these master switches are functionally conserved across different plant species, including woody species.

**Table 1:** Summary of the transcription factors involved in secondary cell wall formation in woody species.

TF group	Species	TF	TF ortholog in *Arabidopsis*	Function	Reference
SWN	*Populus trichocarpa*	PtrSND1-A2 (PtrWND1B)	SND1	Positively regulate fiber cell wall thickening. Its splice variant, PtrSND1-A2^IR^, is a dominant-negative regulator to suppress the transactivation of all PtrSND1 family members.	Li Q. Z. et al., 2012; Zhao et al., 2014
	*Populus trichocarpa*	PtrSND1-B1	SND1	Function as a master regulator to activate a hierarchical gene regulatory network during wood formation.	Lin et al., 2013
	*Eucalyptus grandis*	EgWND1	SND1	Transcriptional activator of SCW biosynthesis in wood.	Zhong et al., 2010a
	*Populus tomentosa* Carr.	PtoVNS11	SND1	Positively regulate lignin deposition and SCW thickening.	Yang et al., 2015
	*Populus trichocarpa*	PtSND2	SND2	Positively regulate fiber SCW thickening and lignin and cellulose biosynthesis.	Wang et al., 2013
	*Populus trichocarpa*	PtrWND2B	NST1/2	Activate SCW TFs and biosynthetic genes.	Zhong et al., 2010b
	*Populus trichocarpa*	PtrWND6B	VND6/7	Activate SCW TFs and biosynthetic genes.	Zhong et al., 2010b
	*Populus trichocarpa*	PtrVND6-C1	VND6	Its splice variant, PtrVND6-C1^IR^, together with PtrSND1-A2^IR^ reciprocally cross-regulate the two TF families.	Lin et al., 2017
	*Pinus pinaster*	PpNAC1	VND6	Positively regulate SCW formation.	Pascual et al., 2018
MYB	*Populus trichocarpa*	PtrMYB3/20	MYB46/83	Activate the biosynthetic pathways of cellulose, xylan and lignin and are directly target of PtrWND2.	McCarthy et al., 2010
	*Eucalyptus gunnii*	EgMYB2	MYB46/83	Positively regulate SCW thickness and activates lignin biosynthetic genes.	Goicoechea et al., 2005
	*Pinus taeda*	PtMYB4	MYB46/83	Bind to AC elements and activate lignin biosynthetic genes.	Patzlaff et al., 2003a
	*Eriobotrya japonica*	EjMYB1	MYB58/63	Bind to AC elements and activate lignin biosynthetic genes.	Xu et al., 2014
	*Populus tomentosa* Carr.	PtoMYB216	MYB61	Positively regulate lignin biosynthetic pathway.	Tian et al., 2013
	*Populus tomentosa* Carr.	PtoMYB170	MYB61	Positively regulate lignin biosynthetic pathway and promote dark-induced stomatal closure.	Xu et al., 2017
	*Pinus taeda*	PtMYB8	MYB61	Positively regulate lignin biosynthetic and other cell wall-related genes.	Bomal et al., 2008
	*Picea glauca*	PgMYB8	MYB61	Positively regulate lignin biosynthesis.	Bedon et al., 2007
	*Populus deltoides*	PdMYB221	MYB4	Repressor. Negatively regulate SCW formation, including cellulose, xylose and lignin.	Tang et al., 2015
	*Populus tomentosa* Carr.	PtoMYB156	MYB4	Repressor. Repress phenylpropanoid biosynthesis and negatively regulate SCW formation.	Yang et al., 2017
	*Eucalyptus gunnii*	EgMYB1	MYB4	Repressor. Negatively regulate SCW formation.	Legay et al., 2007, 2010
	*Leucaena leucocephala*	LlMYB1	MYB4	Repressor. Negatively regulate lignin biosynthesis.	Omer et al., 2013
	*Eriobotrya japonica*	EjMYB2	MYB4	Repressor. EjMYB2 and EjMYB1 competitively interact with AC elements in the promoters of lignin biosynthetic genes.	Xu et al., 2014
	*Populus tremula* L. × *tremuloides* Michx.	PttMYB21a	MYB52	Repressor. Negatively regulate lignin biosynthesis.	Karpinska et al., 2004
	*Quercus suber*	QsMYB1	MYB68	Related to secondary growth and cork biosynthesis.	Almeida et al., 2013
	*Vitis vinifera* L.	VvMYB5a	MYB5	Involved in phenylpropanoid pathway.	Deluc et al., 2006
	*Pinus taeda*	PtMYB1	MYB42/43/20	Bind to AC elements and activate lignin biosynthetic genes.	Patzlaff et al., 2003b
	*Populus trichocarpa*	PtrMYB152	MYB43/20	Positively regulate SCW biosynthesis.	Wang et al., 2014
	*Populus deltoides*	PdMYB10/128	MYB103	Positively regulate fiber SCW thickening and delay flowering.	Chai et al., 2014b
	*Populus deltoides*	PdMYB90/167	MYB52	Negatively regulate fiber and vessel SCW thickening and prompt flowering.	Chai et al., 2014b
	*Populus deltoides*	PdMYB92/125	MYB42	Repressor. Negatively regulate fiber and vessel SCW thickening and prompt flowering.	Chai et al., 2014b
WRKY	*Populus trichocarpa*	PtrWRKY19	WRKY12	Repressor. Negatively regulate pith SCW formation.	Yang et al., 2016
	*Vitis vinifera* L.	VvWRKY2	WRKY3/4	Positively regulate lignin biosynthesis and affect S/G ratio.	Guillaumie et al., 2010
Others:					
HD Zip III	*Populus trichocarpa*	popREVOLUTA (PRE)	REV	Play fundamental roles in cambium initiation and patterning of secondary vascular tissues	Robischon et al., 2011
HD Zip III	*Populus tremula* × *alba*	POPCORONA (PCN)	HB15(CNA)	Involved in SCW lignification and regulate cell differentiation during secondary growth.	Du et al., 2011
HD Zip II	*Eucalyptus camaldulensis*	EcHB1	HAT22(ABIG1)	Negatively regulate lignin and hemicellulose content, increase fiber length and growth.	Sonoda et al., 2009
KNOX I	*Populus tremula* × *alba*	ARBORKNOX1 (ARK1)	STM	Affect internode elongation and secondary vascular cell types in stem, positively regulate lignin biosynthesis.	Groover et al., 2006
KNOX I	*Populus tremula* × *alba*	ARBORKNOX2 (ARK2)	BP	Negatively regulate SCW biosynthesis.	Du et al., 2009
KNOX II	*Populus trichocarpa* × *deltoides*, *P. balsamifera*	PtrKNAT7	KNAT7	Negatively regulate SCW biosynthesis.	Li E. Y. et al., 2012
LBD	*Populus tremula* × *alba*	PtaLBD1	LBD1/11	Positively regulate ray cell development and phloem differentiation.	Yordanov et al., 2010
CCCH	*Populus deltoides*	PdC3H17 and PdC3H18	C3H14	Positively regulate SCW formation and are direct targets of PdMYB3 and PdMYB21.	Chai et al., 2014a
MADS-box	*Populus tremuloides*	PTM5	SOC1	Expressed in differentiating vascular cambium and xylem tissues.	Cseke et al., 2003
ERF	*Populus simonii*? × ? *nigra*	PsnSHN2	SHN2	Positively regulate cellulose and hemicellulose biosynthesis, but negatively regulate lignin biosynthesis.	Liu et al., 2017
EIN	*Populus tremula*	EIN3D	EIN3	Possibly act upstream or together with VIN2 during wood formation.	Seyfferth et al., 2018

## Regulators Associated With the First Layer of Transcription Factors

In the first layer of SCW regulatory network, several TFs are involved in regulation or interaction with the master switches (Figure 1). For instance, VND-INTERACTING2 (VNI2) interacts with VND7 and VND1-5. Here, VNI2 functions as a transcriptional repressor to limit the expression of VND7-regulated vessel-specific genes (Yamaguchi et al., 2010). XYLEM NAC DOMAIN1 (XND1) is up-regulated in xylem, and it can negatively regulate xylem vessel differentiation (Zhao et al., 2008). A recent study indicates the function of XND1 in xylem differentiation depends on its C-terminal region containing linear motifs (KII-acidic, LXCXE, E2F^TD^-like and LXCXE-mimic) which can interact with the cell cycle and differentiation regulator RETINOBLASTOMA-RELATED (RBR) (Zhao et al., 2017). By using enhanced yeast one hybrid assays, Taylor-Teeples et al. (2015) identified E2Fc as a key upstream regulator of VND6, VND7 and other SCW biosynthetic genes. E2Fc is a known negative regulator of endoreduplication (del Pozo et al., 2002, 2006), but it can also act as a transcriptional activator (Kosugi and Ohashi, 2002; Heckmann et al., 2011). Prior to terminal differentiation, the elongating xylem cells likely undergo endoreduplication before SCW deposition via E2Fc-mediated activation or repression of VND7 in a concentration-dependent manner (Taylor-Teeples et al., 2015).

Other regulators associated with master switches in the first layer of SCW regulatory network include Homeobox HD-Zip class III (HD-Zip III), a small TF family that consists of five members in the *Arabidopsis* genome, i.e., REVOLUTA/INTERFASCICULAR FIBERLESS1 (REV/IFL1), PHABULOSA (PHB), PHAVOLUTA (PHV), HB8, and HB15 (CORONA). The HD-Zip III genes are negatively regulated by highly conserved miRNAs (Floyd et al., 2006) and all five HD-Zip III TFs are necessary for xylem cell specification and SCW synthesis. In *Populus*, *popREVOLUTA* (ortholog of *REV*) plays fundamental roles in the initiation of the cambium and in the regulation of the patterning of secondary vascular tissues (Robischon et al., 2011). The promoters of *REV* and *PHB* can be bound and regulated by VND7 (Taylor-Teeples et al., 2015). HB15 is necessary for repressing SCW biosynthesis in pith and disruption of the expression of *HB15* causes ectopic lignification in pith cells. An ortholog in *Populus*, *POPCORONA*, is involved in SCW lignification and regulates cell differentiation during secondary vascular growth (Du et al., 2011). Noticeably, the expression of *WRKY12* is up-regulated in *athb15* mutant (Du et al., 2015). As a negative regulator, WRKY12 can directly bind to the *NST2* promoter to repress its expression, thus repressing the SCW thickening in pith cells (Wang et al., 2010). Finally, three homologous LOB domain TFs (LBD15, LBD18, and LBD30) are expressed in differentiating TEs and enhance the transcription of *VND7* in a positive feedback loop (Soyano et al., 2008; Ohashi-Ito et al., 2018).

In addition to the transcriptional regulation, the post-translational modifications play important roles in regulating the master switches in the first layer of SCW regulatory network. A study using tobacco BY-2 cells expressing VND7-YFP together with the treatment of proteasome inhibitor MG-132 showed that VND7 is also regulated by proteolysis (Yamaguchi et al., 2008). Recently, Kawabe et al. (2018) identified a recessive mutant with inhibited ectopic xylem cell differentiation in *35S::VND7-VP16-GR* lines and found this mutant is caused by a single amino acid substitution (E36K) in S-nitrosoglutathione reductase (GSNOR1). GSNOR was first reported as a glutathione-dependent formaldehyde dehydrogenase and regulates the turnover of *S*-nitrosoglutathione, a natural nitric oxide donor. VND7 can be *S*-nitrosylated at Cys264 and Cys320, which are located near the transactivation domain. The *in vivo S*-nitrosylation of VND7 mediated by GSNOR1 affects VND7-downstream signaling events and thereby leading to deficient xylem vessel differentiation (Kawabe et al., 2018). Collectively, these regulators work with the first layer master switches to regulate their transcription or protein activity by providing post-translational modifications. These provide an additional layer of regulation at the top level to regulate SCW biosynthesis, which may possibly involve the integration of developmental or environmental signals since many of these regulators play roles in these processes (Jin et al., 2000; Preston et al., 2004; Romano et al., 2012).

## The Second Layer of Transcription Factors in the Regulatory Network in SCW Formation

A series of additional TFs make up the second layer of regulation of the expression of SCW biosynthetic genes and other downstream genes. The master switches in the second layer are MYB46 and MYB83 (Figure 1), which are directly regulated by SND1 and its close homologs (NST1, NST2, VND6 and VND7) (Zhong et al., 2007; McCarthy et al., 2009). *MYB46* and *MYB83* are functional redundant and are specifically expressed in fibers and vessels where SCW thickening occurs. Overexpression of *MYB46* or *MYB83* enhanced the biosynthetic pathways of lignin, cellulose and xylan, and resulted in ectopic deposition of SCW; whereas RNAi or dominant repression of *MYB46* and *MYB83* reduced SCW thickening of fibers and vessels (Zhong et al., 2007; McCarthy et al., 2009).

MYB46 and MYB83 can regulate other SCW-related TFs or directly regulate the SCW structural genes. Based on results from the estrogen-inducible direct activation system, several downstream TFs, including *MYB43*, *MYB52*, *MYB54*, *MYB58*, *MYB63* and *KANT7*, have been identified as direct targets of MYB46/83. A 7-bp sequence ACC(A/T)A(A/C)(T/C) has been designated as the secondary wall MYB-responsive element (SMRE) (Zhong and Ye, 2012), similar to binding sequences of AC element [ACC(T/A)ACC] (Fornale et al., 2010) and P1 [CC(T/A)ACC] (Grotewold et al., 1994). Another 8-bp sequence [(T/C)ACC(A/T)A(A/C)(T/C)] has also been identified as MYB46 specific binding sequence, namely MYB46-responsive *cis*-regulatory element (M46RE) (Kim et al., 2012; Ko et al., 2014). In addition, MYB46/83 can directly regulate SCW structural genes. For example, MYB46 directly regulates all three SCW-associated *cellulose synthase* genes (*CesA4*, *CesA7* and *CesA8*) (Kim et al., 2013) and a *mannan synthase CSLA9* (Kim et al., 2014b). Noticeably, the promoters of these genes contain multiple M46REs. A genome-wide screen of promoter sequences indicates the xylan biosynthetic genes (*IRX8*, *IRX9*, *IRX10*, *IRX14, IRX15* and *IRX15-L*) (Jensen et al., 2011; Kim et al., 2014a), lignin biosynthesis-related laccase (*LAC4/IRX12*, *LAC10* and *LAC11*) (Zhao et al., 2013), cytoskeleton-related genes (*Myosin5*, *microtubule-associated protein*), and homologous of *IRX15/15-L* (*DUF579s*) also contain multiple M46REs in their promoter regions (Jensen et al., 2011).

Similar to the master switches in the first layer of SCW regulatory network, the function of MYB46 and MYB83 is also highly conserved in woody species. For instance, PtrMYB3 and PtrMYB20 from *Populus*, EgMYB2 from *Eucalyptus*, and PtMYB4 from *Pinus taeda*, are orthologs of MYB46/83 and perform the same function as MYB46/83 from *Arabidopsis* in SCW biosynthesis (Figure 2). In *Populus* developing wood, *PtrMYB3* and *PtrMYB20* are highly expressed in vessels and fibers and can regulate the biosynthesis of lignin, cellulose and xylan (McCarthy et al., 2010). *Eucalyptus EgMYB2* is identified based on a quantitative trait locus (QTL) for lignin content. EgMYB2 can specifically bind to the promoters of lignin biosynthetic genes, such as *CINNAMOYL-COENZYME A REDUCTASE* (*CCR*) and *CINNAMYL ALCOHOL DEHYDROGENASE* (*CAD*). Overexpression of *EgMYB2* enhanced SCW thickness in transgenic tobacco (Goicoechea et al., 2005). In loblolly pine, *Pinus taeda MYB4* (*PtMYB4*), the homolog of *Arabidopsis MYB46*/*83*, is expressed in lignificating xylem cells. PtMYB4 can bind to AC elements and activate the expression of target genes (Patzlaff et al., 2003a). Collectively, these results suggest that the orthologs of MYB46/83 function conservatively as the second layer master regulators in SCW biosynthesis in woody plants.

## The Third Layer of Regulatory Network in SCW Formation

In addition to the master switches in the second layer of SCW regulatory network, there are TFs that regulate SCW biosynthesis, whose expression are regulated by the master switches MYB46/83, and act as downstream TFs in the third layer of SCW regulatory network (Figure 1). Most of these TFs belong to the MYB gene family. The first identified lignin-specific TFs were *MYB58*, *MYB63*, and *MYB85* (Zhong et al., 2008; Zhou et al., 2009). Most monolignol biosynthetic genes contain AC elements in their promoter region and are direct target of MYB58 (Zhou et al., 2009). Moreover, *MYB6*, *MYB20*, *MYB42*, *MYB43*, *MYB52*, *MYB54*, *MYB61*, *MYB103*, etc. are also developmentally associated with cells undergoing SCW thickening (Zhong et al., 2008; Romano et al., 2012). *MYB52*, *MYB54*, *MYB85* and *MYB103* are able to induce SCW biosynthetic genes. Overexpression of *MYB85* led to ectopic lignin deposition in epidermal and cortical cells; overexpression of *MYB103* increased SCW thickening in fibers; whereas dominant repression of *MYB52*, *MYB54*, *MYB85*, or *MYB103* reduced SCW thickening in fiber cells (Zhong et al., 2008). In contrast, *MYB61* plays multiple regulatory roles in plant development, including lignification, dark-photomorphogenesis (Newman et al., 2004), stomatal aperture (Liang et al., 2005) and seed coat mucilage deposition (Penfield et al., 2001). Analysis of loss-of-function mutant of *MYB61*, *atmyb61*, showed that MYB61 can activate the expression of *CAFFEOYL-COA 3-O-METHYLTRANSFERASE* (*CCoAOMT*) and *PECTIN METHYLESTERASE* (*PME*) and affect xylem formation and xylem cell structure (Romano et al., 2012).

While most of these TFs activate the expression of their targets and positively regulate SCW biosynthesis, several members in MYB family play negative roles in SCW biosynthesis. *Arabidopsis MYB4* is induced by UV-B. Overexpression of *MYB4* can repress the transcription of *4CL*, *C4H* and *CAD* in tobacco (Jin et al., 2000). *MYB7* and *MYB32* share high sequence similarity with *MYB4*, act as repressors, and are strongly activated by MYB46 (Ko et al., 2009). MYB32 negatively regulates lignin pathway through repressing other targets, such as *COMT* (Preston et al., 2004). In addition, there is a feedback regulation between MYB32 and SWNs. The transcription of *MYB32* is repressed in the *nst1 nst3* double mutant (Mitsuda et al., 2007). A later study based on *in vitro* trans-activation assays and electrophoretic mobility shift assay (EMSA) further confirmed that *MYB32* is directly regulated by SND1 (Wang et al., 2011). Furthermore, *SND1* is negatively regulated by MYB32 (Wang et al., 2011), implying that both positive and negative feedforward loop exist in SCW regulatory network.

*Populus PttMYB21a*, a homolog of *MYB52* (Figure 2), can negatively regulate the expression of *CCoAOMT* and the acid soluble lignin content (Karpinska et al., 2004). In grapevine, *VvMYB5a* can regulate both anthocyanin/proanthocyanidin biosynthesis and lignin biosynthesis (Deluc et al., 2006). In *Eucalyptus gunnii*, EgMYB1 binds to the promoter of *CCR* and *CAD* to repress the monolignol biosynthesis (Legay et al., 2007). In *Pinus taeda, PtMYB1*, closely related to *Arabidopsis MYB42*, *MYB43* and *MYB20*, is most abundantly expressed in differentiating xylem and functions as a transcriptional activator through binding the AC elements (Patzlaff et al., 2003b). Loquat (*Eriobotrya japonica*) EjMYB1 (ortholog of MYB58 and MYB63) functions as transcriptional activator and can activate both *Arabidopsis* and loquat lignin biosynthetic genes. EjMYB2 (ortholog of MYB4) functions as a repressor and can counter the induction by EjMYB1 (Xu et al., 2014). The large abundance of TFs in the third layer provide multiple opportunities to connect to the master switches in the first and second layers and the structural genes in SCW biosynthesis, and to fine tuning the pathways.

## Regulators Associated With the Third Layer of Transcription Factors

Several genes in other TF families cooperate with MYBs or act independently to regulate the SCW biosynthesis (Figure 1). KNOTTED ARABIDOPSIS THALIANA7 (KNAT7) is a Knotted-like homeobox (KNOX) protein, is a target of MYB46 (Ko et al., 2009) and SND1 (Zhong et al., 2008), and can also be regulated by MYB61 (Romano et al., 2012). Dominant repression of *KNAT7* reduced SCW thickening in vessels and fibers (Zhong et al., 2008). In *Nicotiana*, virus-induced silence and RNAi of *NbKNAT7* inhibited the thickening of fiber cell walls and repressed the expression of lignin, cellulose and xylan biosynthetic genes (Pandey et al., 2016). KNAT7 was known as a transcriptional repressor that negatively regulates SCW biosynthesis, and it can physically interact with MYB75, OFP4 and BLH6 (Li et al., 2011; Li E. Y. et al., 2012; Bhargava et al., 2013; Liu et al., 2014). *Arabidopsis* MYB75 positively regulates anthocyanin biosynthesis, but it functions as a repressor in SCW biosynthesis. A loss-of-function mutant *myb75-1* enhanced the expression of lignin, cellulose and xylan biosynthetic genes and increased SCW thickness in xylary and interfascicular fibers (Bhargava et al., 2010). In *Arabidopsis*, the KNAT7-MYB75 complex is involved in modulating SCW formation in both inflorescence stem and seed coat (Bhargava et al., 2013). OFP4 is an Ovate Family Protein transcriptional co-regulator and can interact with KNAT7 and enhance the repression activity of KNAT7 in SCW biosynthesis (Li et al., 2011). BLH6 is a BELL1-LIKE HOMEODOMAIN protein and functions as a transcriptional repressor. It specifically interacts with KNAT7 to enhance its repression activity. BLH6 and KNAT7 can repress the expression of *REV* through directly binding to its promoter (Liu et al., 2014).

In *Populus*, *KNAT7* functions as a repressor in a negative feedback loop in SCW formation (Li E. Y. et al., 2012). However, a recent study indicates that KNAT7 positively regulates xylan biosynthesis through directly activating the expression of IRX9 (He et al., 2018). Another member in KNOX family, *BERVIPEDICELLUS* (*BP*)/*KNAT1*, also plays a role in the lignin pathway. BP binds to the promoters of genes in the lignin pathway (*COMT*, *CCoAOMT*, etc.) and overexpressing *BP* significantly decreases the SCW lignification (Mele et al., 2003). In addition, the tandem CCCH zinc finger (TZF) TF, *C3H14*, is able to activate SCW biosynthetic genes and is directly regulated by MYB46 and SND1 (Ko et al., 2009). Its orthologs in *Populus deltoides*, *PdC3H17* and *PdC3H18*, also positively regulate SCW formation in both *Populus* and *Arabidopsis*, and are direct targets of PdMYB3 and PdMYB21 (Chai et al., 2014a). These regulators associated with the third layer of transcription factors provide opportunities for fine tuning SCW biosynthesis at the very downstream level.

## Ethylene Related Tfs in SCW Biosynthesis

Recently, a class of ethylene signaling-related TFs have attracted the attention of researchers due to their function in wood development (Figure 1). Ethylene is the smallest phytohormone with the simple structure C_2_H_4_, and is involved in various plant developmental processes including leaf development, senescence, fruit ripening, germination, stress responses, etc. (Dubois et al., 2018). Notably, ethylene is also involved in multiple process during wood formation, including cambial growth, xylem cell morphogenesis, and vessels/fibers/rays ontogenesis (Little and Savidge, 1987). In angiosperm trees, ethylene, as an important signaling molecule, is involved in the remodeling of wood formation upon tension wood induction. Exogenous application of ethylene or its precursor 1-aminocyclopropanel-1-carboxylic acid (ACC) enhances xylem growth in hybrid aspen (*Populus tremula* × *P. tremuloides*) (Love et al., 2009). In addition, gene expression and enzyme activity of ACC oxidase are up-regulated on the tension wood surface (Andersson-Gunneras et al., 2003).

The ethylene perception and signal transduction cascades depend on ethylene-induced Ethylene Response Factors (ERFs). In *Arabidopsis*, *ERF1*, *ERF018* and *ERF109* are involved in the vascular cell division (Etchells et al., 2012), suggesting that ERFs-mediated ethylene signaling is important for vascular development. Vahala et al. (2013) performed a genome-wide screen for *ERFs* in hybrid aspen stem. Among 170 *ERFs* in *Populus*, 50 *ERFs* were induced greater than five-fold by ethylene. During tension wood formation, 17 and 8 *ERFs* were induced greater than two-fold and ten-fold, respectively. Subsequently, the function of these ERFs was further confirmed in transgenic *Populus* (Vahala et al., 2013). Overexpression of *ERF18*, *ERF21*, *ERF30*, *ERF85* and *ERF139* in wood-forming tissues modified the wood chemotype in hybrid aspen. Overexpression of *ERF139* repressed longitudinal and lateral growth with altered wood development, overexpression of *ERF18*, *ERF34*, and *ERF105* enhanced diameter growth, whereas overexpression of *ERF71* and *ERF85* suppressed diameter growth.

Despite this work the role of ERFs-mediated ethylene signaling in the SCW regulatory network remains elusive (Figure 1). Liu et al. (2017) reported that *Populus* ERF type TF, *PsnSHN2*, is predominantly expressed in xylem tissues, and that it positively regulates cellulose and hemicellulose biosynthesis but negatively regulates lignin biosynthesis. Recently, Seyfferth et al. (2018) constructed an ethylene-related gene expression network during SCW formation, *ETHYLENE INSENSITIVE 3D* (*EIN3D*) and 11 *ERFs* were identified as hub genes. Interestingly, a *VNI2* homolog is highly associated with *EIN3D*, suggesting EIN3D may act upstream or together with VIN2 during SCW formation. How to precisely position these unresolved TFs into the SCW transcriptional regulatory network deserves further investigation.

## Post-Transcriptional Regulation of Tfs Involved in SCW Formation

The activity of transcriptional regulators and the gene expression are also affected by post-transcriptional regulation. Alternative splicing is an important model of post-transcriptional regulation. It plays important roles for enhancing proteomic diversity in diverse cellular processes (Chen and Manley, 2009). In plants, more than 60% of intron-containing genes undergo alternative splicing (Syed et al., 2012). However, the knowledge of alternative splicing in wood formation is limited. By analyzing the xylem transcriptome in 20 *P. trichocarpa* genotypes, Bao et al. (2013) found that about 36% of the genes expressed in xylem undergo alternative splicing, especially those cell wall biosynthetic genes including glycosyl transferases and C_2_H_2_ TFs.

Interestingly, most key TFs in the first layer of SCW regulatory network undergo alternative splicing. In *Populus*, a “stem-differentiating xylem”-specific variant of *SND1*, *PtrSND1-A2^IR^*, was identified as a dominant-negative regulator of SND1-mediated pathway (Li Q. Z. et al., 2012). The retained intron 2 in *PtrSND1-A2^IR^* cDNA introduces a premature stop codon resulting in a truncated protein lacking the activation domain. Hence PtrSND1-A2^IR^ loses DNA binding and transactivation abilities, and it represses the transcription of *PtrSND1* members and *PtrMYB021* via its retained dimerization capability. This is the first report on the auto-repression of a TF family by its own splice variant in plants. Subsequently, Zhao et al. (2014) compared the function of the two isoforms, *PtrSND1-A2* (also named *PtrWND1B-s*) and *PtrSND1-A2^IR^* (also named *PtrWND1B-l*), during wood formation. Overexpression of *PtrWND1B-s* or *PtrWND1B-l* oppositely regulate fiber SCW thickening in *Populus*. This alternative splicing type was also detected in *SND1* ortholog in *Eucalyptus grandis* (*Eucgr.E01053*), but not in *Arabidopsis*, implying that the alternative splicing regulation of *SND1* may be different between woody species and herbaceous plants (Li Q. Z. et al., 2012; Zhao et al., 2014). Recently, Lin et al. (2017) reported that another key TF in the first layer of SCW regulatory network, *VND6*, also undergoes alternative splicing during wood formation. Its splice variant retained intron 2, *PtrVND6-C1^IR^*, which suppresses the protein function of all *PtrVND6* and *PtrSND1* family members, including *PtrSND1-A2*. In addition, *PtrVND6-C1* can also be suppressed by *PtrSND1-A2^IR^*. *PtrVND6-C1^IR^* and *PtrSND1-A2^IR^* function together for reciprocal cross-regulation of VND and SND members to maintain homeostasis for xylem differentiation and plant development. Whether other key TFs in SCW regulatory network also undergo alternative splicing is still an open question. This intron-retained splice variant-introduced reciprocal cross-regulation provides an additional insight for studying the regulation mechanism of SCW formation and appears to be woody species-specific.

In addition to alternative splicing, the TFs and structural genes in SCW regulatory network are regulated by non-coding RNAs (ncRNAs). In the past few decades, ncRNAs have been shown to play key regulatory roles in various biological processes of development and stress response (Mallory and Vaucheret, 2006; Wierzbicki, 2012). Plant ncRNAs can be classified into various types according to their molecular structures, including microRNA (miRNA), small interfering RNA (siRNA), long ncRNA (lncRNA), circular RNA (circRNA), etc. (Sunkar et al., 2007; Kim and Sung, 2012). Here, we focus on the role of miRNA and lncRNA in SCW formation, in particular on their regulation of SCW-related TFs. Lu et al. (2005) identified 21 miRNA families from the developing xylem of *P. trichocarpa* stems. Among them, 11 miRNA families have conserved sequences in *Arabidopsis* but exhibit species-specific developmental expression patterns, while 10 *Populus*-specific miRNA families might be involved in tree-specific processes. Several members in miRNA families have been reported to play important roles in SCW formation. miRNA165/166 are known to target HD-Zip III TFs, and control xylem differentiation through modulating the *PHB* gradients in the stele to maintain *PHB* at a low dosage in protoxylem and a high dosage in metaxylem differentiation (Carlsbecker et al., 2010; Miyashima et al., 2011). In hybrid aspen (*Populus tremula* × *P. alba*), *Pta-miRNA166* targets *PtaHB1*, a homolog of *REV*, to regulate secondary growth (Ko et al., 2006). In a gain-of-function *Arabidopsis MIR166a* mutant, the transcript level of *HB15* was decreased and xylem and interfascicular region were expanded in vascular tissue (Kim et al., 2005). In *Populus*, synthetic miRNA knock-down of *POPCORONA* (*PCN*), an ortholog of *HB15*, disturbed the lignification of pith cells, whereas overexpression of a miRNA-resistant *PCN* delayed the lignification of xylem and phloem fibers (Du et al., 2011). Laccases (LAC) belong to the blue copper oxidase family and polymerize monolignols into lignin. Among the 49 *LAC* genes in the *Populus* genome, 29 were predicted as the targets of *ptr-miRNA397a*. Overexpression of *Ptr-MIRNA397a* reduced lignin content without changing monolignol biosynthesis in *Populus* (Lu et al., 2013). Recently, another miRNA, *miRNA319*, was also shown to be able to target *TCP4* and decrease the SCW formation in *Arabidopsis* stem. TCP4 TF can directly activate the expression of *VND7* via binding to its promoter (Sun et al., 2017). lncRNAs are also involved in wood formation. Chen et al. (2015) performed a genome-wide analysis and compared the expression profiles of lncRNA in the xylem of normal wood, opposite wood and tension wood in *Populus tomentosa.* A total of 16 genes in cellulose or lignin biosynthetic pathways were targeted by lncRNAs. Combining whole-genome resequencing with growth and wood-property traits of 435 *P. tomentosa* individuals, Zhou et al. (2017) further identified 8 lncRNAs and 15 potential target genes in the phenylpropanoid pathway. These diversified post-transcriptional regulatory mechanisms offer new perspectives to the SCW regulatory network through modifying gene expression or protein diversity of the key TFs.

## Conclusion

In this review, we provide a summary of current knowledge of the transcriptional regulation of SCW biosynthesis in woody species and contrast to what is known in other plant species, particularly in the model plant *Arabidopsis*. Woody species and the herbaceous model plant *Arabidopsis* share conserved master switches in the SCW transcriptional regulatory network, especially in the first and second layers of the network. However, the large abundance of TFs in the third layer and diversified post-transcriptional regulatory mechanisms make the SCW regulatory network more complex in woody plants. For example, the alternative splicing events of SND and VND genes appeared to be woody species-specific. This poses more challenges for fully revealing the SCW regulatory mechanism in woody species. Recent advances in high-throughput sequencing provide great potentials for improving the genome annotation and identifying alternative splicing events and lncRNAs during SCW formation. In addition, expression quantitative trait loci (eQTL) analysis provides an effective and efficient way to identify novel regulators, especially in tree species with long life cycle. Recently, Zhang et al. (2018) identified a *Populus* hydroxycinnamoyl-CoA:shikimate hydroxycinnamoyl transferase PtHCT2 controlling caffeoylquinic acid biosynthesis and its upstream regulators through eQTL analysis, which provides a new strategy to identify novel transcriptional regulators in woody plants. Considering the ecological and economic values of woody species, it is important to understand the woody species-specific transcriptional regulation of SCW formation, and this is a fruitful area for further research.

## Author Contributions

JZ collected and synthesized data from literature and wrote the manuscript. MX, GT, WM and J-GC revised the manuscript.

## Conflict of Interest Statement

The authors declare that the research was conducted in the absence of any commercial or financial relationships that could be construed as a potential conflict of interest.
